# The Peopling and Migration History of the Natives in Peninsular Malaysia and Borneo: A Glimpse on the Studies Over the Past 100 years

**DOI:** 10.3389/fgene.2022.767018

**Published:** 2022-01-27

**Authors:** Boon-Peng Hoh, Lian Deng, Shuhua Xu

**Affiliations:** ^1^ Faculty of Medicine and Health Sciences, UCSI University, UCSI Hospital, Port Dickson, Malaysia; ^2^ State Key Laboratory of Genetic Engineering, Center for Evolutionary Biology, Collaborative Innovation Center for Genetics and Development, School of Life Sciences, Fudan University, Shanghai, China; ^3^ Key Laboratory of Computational Biology, Shanghai Institute of Nutrition and Health, University of Chinese Academy of Sciences, Chinese Academy of Sciences, Shanghai, China; ^4^ Department of Liver Surgery and Transplantation Liver Cancer Institute, Zhongshan Hospital, Fudan University, Shanghai, China; ^5^ Ministry of Education Key Laboratory of Contemporary Anthropology, School of Life Sciences, Human Phenome Institute, Fudan University, Shanghai, China; ^6^ School of Life Science and Technology, ShanghaiTech University, Shanghai, China; ^7^ Jiangsu Key Laboratory of Phylogenomics and Comparative Genomics, School of Life Sciences, Jiangsu Normal University, Xuzhou, China; ^8^ Henan Institute of Medical and Pharmaceutical Sciences, Zhengzhou University, Zhengzhou, China; ^9^ Center for Excellence in Animal Evolution and Genetics, Chinese Academy of Sciences, Kunming, China

**Keywords:** Orang Asli, natives, admixture, divergence, peopling history, migration

## Abstract

Southeast Asia (SEA) has one of the longest records of modern human habitation out-of-Africa. Located at the crossroad of the mainland and islands of SEA, Peninsular Malaysia is an important piece of puzzle to the map of peopling and migration history in Asia, a question that is of interest to many anthropologists, archeologists, and population geneticists. This review aims to revisit our understanding to the population genetics of the natives from Peninsular Malaysia and Borneo over the past century based on the chronology of the technology advancement: 1) Anthropological and Physical Characterization; 2) Blood Group Markers; 3) Protein Markers; 4) Mitochondrial and Autosomal DNA Markers; and 5) Whole Genome Analysis. Subsequently some missing gaps of the study are identified. In the later part of this review, challenges of studying the population genetics of natives will be elaborated. Finally, we conclude our review by reiterating the importance of unveiling migration history and genetic diversity of the indigenous populations as a steppingstone towards comprehending disease evolution and etiology.

## Introduction

Southeast Asia (SEA) is believed to be one of the earliest regions of hominin habitation recorded outside Africa nearly 2 million years ago, following the arrival of the ancient “Java Man” known as the *Homo erectus* (Jin et al., 2001). Archeological evidence from sites such as Tam Pa Ling in Laos (Demeter et al., 2012), Callao Cave in the Philippines (Mijares et al., 2010), and Niah Cave in Borneo Malaysia (Barker et al., 2007; Curnoe et al., 2016), suggest that SEA may have occupied by anatomically modern humans (AMH) at least 50–70 thousand years ago (kya). Today this landmass is home to ∼600 million people, enriched with cultural, linguistic, and genetic diversity.

SEA was once a great landmass bridging the Eurasia during the last glacial maximum peaked ∼20 kya (Bellwood, 2007). This landmass, called the Sundaland, linked the present day Peninsular Malaysia and Borneo, the southern Philippines, and the west and south Indonesia islands. Subsequently, the Sundaland was hit by three great sea-level surges in approximately 14, 11, and 8 kya, respectively. The sea level rose by 120 m, thus forming the present-day map of SEA. These climate and geographical changes had drastically changed the flora and fauna in SEA, and plausibly the prehistorical peopling of modern human to the land of SEA (Macaulay et al., 2005; Bellwood, 2007; HUGO Pan-Asian SNP Consortium et al., 2009; Arenas et al., 2020).

**FIGURE 1:**
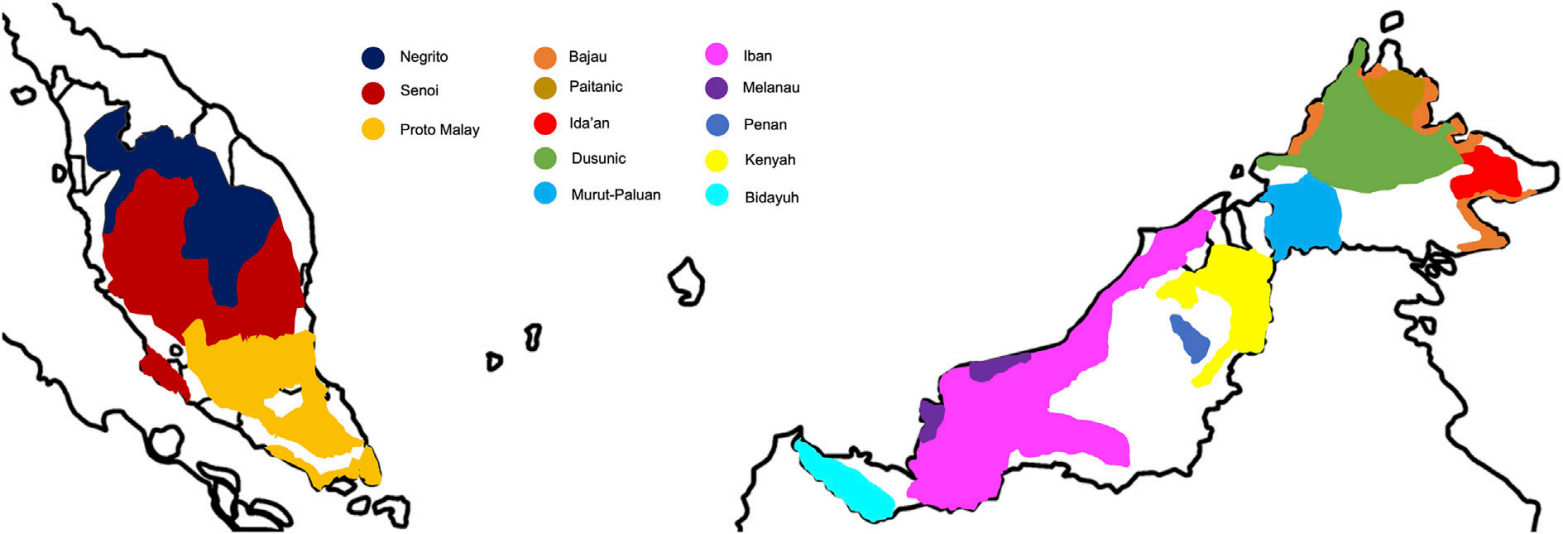
Distribution of the major native populations from Peninsular Malaysia and Borneo.

Malaysia has been an important piece of puzzle to the global map of peopling and migration history, owing to its strategic location at the crossroad of the mainland and islands of SEA. It is a multi-ethnic, multi-lingual, and multi-cultural nation with diverse socio-economic practices. The Malay ethnic group is the predominant native in Malaysia, making up ∼61% of the total population; whereas the natives from Borneo comprise ∼9% of the total population (Figure 1). The indigenous populations from Peninsular Malaysia, locally known as the Orang Asli, constitute ∼0.5% of the total population. They are heterogeneous and are categorized into Negrito, Senoi, and Proto Malay, each sub-classified into different sub-tribes (Table 1). Traditionally, the Negritos are hunters-gatherers that practice egalitarianism, semi-nomadic and patrilineal descent system. The Senoi was primitive slash-and-burn farmers, and the Proto-Malays are primarily agriculturists and fishermen.

**TABLE 1:** Classification of Orang Asli and some major sub-ethnic populations from Borneo. Each Orang Asli group is further divided into six sub-tribes. The natives Sabahan are primarily classified into five language groups (Dusunic, Paitanic, Murutic, Ida’anic, and Sama-Bajaw). The major Sarawak natives include the two major communities namely, the Dayaks and the Orang Ulu.

Geographical distributions	Group	Sub-tribe	Language family (Aslian language branch)	Traditional socioeconomic activity
Northern Peninsular Malaysia	Negrito	Bateq	Austroasiatic (Northern Aslian)	Hunting and gathering
		Mendriq	Austro-Asiatic (Northern Aslian)	Hunting and gathering
		Jehai	Austroasiatic (Northern Aslian)	Hunting and gathering
		Kensiu	Austroasiatic (Northern Aslian)	Hunting and gathering
		Kintak	Austroasiatic (Northern Aslian)	Hunting and gathering
		Lanoh	Austroasiatic (Central Aslian)	Swine-and-burn; hunting and gathering
Central Peninsular Malaysia	Senoi	Semai	Austroasiatic (Central Aslian)	Swine-and-burn
		Temiar	Austroasiatic (Central Aslian)	Swine-and-burn
		Che Wong	Austroasiatic (Northern Aslian)	Swine-and-burn; hunting and gathering
		Mah Meri	Austroasiatic (Southern Aslian)	Swine-and-burn; hunting and gathering
		Semaq Beri	Austroasiatic (Southern Aslian)	Swine-and-burn
		Jahut	Austroasiatic (Central Aslian)	Swine-and-burn
Central- to Southern Peninsular Malaysia	Proto-Malay	Jakun	Austronesian	Farming and fishing
		Temuan	Austronesian	Agriculture
		Semelai	Austroasiatic (Southern Aslian)	Swine-and-burn
		Orang Kanaq	Austronesian	Agriculture
		Seletar	Austronesian	Fishing; hunting and gathering
		Orang Kuala	Austronesian	Fishing
West coast Sabah	Dusunic	Dusun	Austronesian	Agriculture
West coast Sabah		Kadazan	Austronesian	Agriculture; hunting and gathering
Northern Sabah		Rungus	Austronesian	Agriculture
Northwest coastal Sabah		Bisaya	Austronesian	Agriculture
Northern Sabah		Bonggi	Austronesian	Agriculture; fishing
West coast Sabah		Lotud	Austronesian	Agriculture
Northern Sabah		Kimaragang	Austronesian	Agriculture; fishing
Western coastal Sabah		Tatana	Austronesian	Agriculture; fishing
East coast Sabah		Minokok	Austronesian	Agriculture
Northern Sabah		Sonsogon	Austronesian	Agriculture; hunting and gathering
Northeast coast Sabah	Paitanic	Sungai	Austronesian	Agriculture; fishing
Northern Sabah		Lingkabau	Austronesian	Agriculture; fishing
Southwestern Sabah	Murutic	Murut	Austronesian	Agriculture; fishing; hunting-and-gathering
		Paluan	Austronesian	Agriculture; fishing; hunting-and-gathering
		Tagal	Austronesian	Agriculture; fishing; hunting-and-gathering
		Selungai	Austronesian	Agriculture; fishing; hunting-and-gathering
East coast Sabah	Ida’anic	Ida’an	Austronesian	Hunting-and-gathering
		Begahak	Austronesian	Agriculture; fishing; hunting-and-gathering
		Subpan	Austronesian	Agriculture; fishing; hunting-and-gathering
East coast Sabah	Sama-Bajaw	Bajau	Austronesian	Seafaring
Throughout Sarawak state	Dayak	Iban (Sea Dayak)	Austronesian	Seafaring; agriculture
Southwest Sarawak (adjacent to West Kalimantan)		Bidayuh (Land Dayak)	Austronesian	Agriculture
Northeast Sarawak	Orang Ulu	Kelabit	Austronesian	Agriculture
Interior areas of Sarawak		Penan	Austronesian	Hunting-and-gathering
Interior areas of Sarawak		Kenyah	Austronesian	Swine-and-burn; hunting-and-gathering; fishing
Interior areas of Sarawak		Kayan	Austronesian	Agriculture; fishing
Northern Sarawak		Lun Bawang; or Lundayeh	Austronesian	Fishing; agriculture; hunting-and-gathering
Northern Sarawak		Bisaya	Austronesian	Agriculture
Interior areas of Sarawak		Sebop	Austronesian	Agriculture
Coastal area of central Sarawak	Melanau		Austronesian	Agriculture; fishing
Interior areas of Sarawak	Punan		Austronesian	Swine-and-burn
Southwest Sarawak (adjacent to West Kalimantan)	Kedayan		Austronesian	Agriculture; fishing

The Malaysian section of Borneo comprises the states of Sabah and Sarawak. The Sabah state (formerly known as North Borneo) is home to culturally diverse populations, comprising of more than 40 ethnicities that are broadly categorized into five major groups: Dusunic, Paitanic, Murutic, Ida’anic, and Sama-Bajaw (Combrink et al., 2008), with Kadazandusun being the largest ethnicity. Most of the native Sabahan inhabit the inland of northern Borneo. Traditionally, they were agriculturists and hunter-gatherers, except for a few populations scattering along the coastal lines of northern Borneo who are seafarers (mainly the Bajaus). The Ida’anic populations used to practice hunting and gathering as their subsistence. The Muruts were once head-hunters but the practice has been ceased during the British colonization. The Sarawak native population consists of some 40 ethnicities. They are either farmers or fishermen; whereas some rely on forest resources as their subsistence (e.g., Penan). The Iban (also known as Sea Dayak) is the major ethnic group, follow by Bidayuh (or known as Land Dayak), Melanau, and many others (Table 1). Like the Muruts, the Iban were formerly reputed as the head-hunters. They were seafarers or agriculturists. The Sarawak natives are believed to have migrated from the neighboring islands of SEA (e.g., Bisaya are thought to be linked with the Visayan of the Philippines) (Williams, 1965; Ibrahim et al., 1985); whereas some have little historical evidence regarding their origin (e.g., the Kenyah and Punan Bah tribes) (Nicolaisen, 1976). The ethnicities of the Sabah and Sarawak are primarily distinguished by their linguistic and sociocultural practices, and has been a long-standing debate.

The SEA populations are enriched with their linguistics spectrum, primarily represented by five language families: Austroasiatic, Austronesian, Tai-Kadai, Hmong-Mien and Sino-Tibetan (Wang, 2001; Enfield, 2005). Nonetheless the natives from Malaysia speak languages that fall under either Austroasiatic, or Austronesian families. The Negrito and Senoi tribes basically speak the “Aslian” language that belongs to the Mon-Khmer linguistic branch that falls under Austroasiatic family, each tribe has their own dialects; whereas the rest of the populations are primarily categorized as the Austronesian language speaking family. The Proto-Malay and the modern Malays speaks the language that derived from the Malayan group; while the Borneo natives’ languages fall under the Malayo-Polynesian group.

Given such rich ethnological diversity and complex prehistorical events in this landmass, addressing migration and peopling history of Peninsular Malaysia and Borneo is therefore a pertinent question.

In this article, we attempt to summarize an overview on our understanding of migration and peopling histories of the natives from Peninsular Malaysia and Borneo over the past century, based on five chronological eras of genomic technological advancement: 1) Anthropological and Physical Characterization; 2) Blood Group Markers; 3) Protein Markers; 4) Mitochondrial and Autosomal DNA Markers; and 5) Whole Genome Analysis. We also provide some hints to the “missing puzzles” of the peopling history in SEA yet to be uncovered, and finally reiterate the implications of migration and peopling history of SEA populations on human health and diseases.

### Anthropological and Physical Characterization

The study of human populations in Malaysia began in the 19th century when the colonial scholars attempted to classify “race”. Since genetic knowledge was rather limited, hypotheses were built merely based on physical and anthropological observations. The population of the Peninsular Malaysia was first grossly classified into two geographical races: “Negrito-like” and “East Asian-like” (Crawfurd, 1820). Populations originated from the Malay Peninsula were firstly categorized into the “black” and the “brown”, but was refined by John Anderson as the “Negrito” and “non-Negrito” groups, respectively. The non-Negrito group includes, for instance, the “Sakei”, “Orang Bukit” (means “People of the Hill”), and “Orang Laut” (means “People of the Sea”). Anderson considered the Malays as the “native” but not “indigenous” people (Anderson, 1824). These classifications were primarily rationalized by outward physical attributes including skin pigmentation (dark, brown light), hair morphology (woolly, wavy, straight, brownie, black), eye color, and stature (tall, short). Other considerations included language, culture, and subsistence (forager, farmer). These physical characteristics are still very much in use to date.

The basic “three-way division” framework was raised by the British anthropologists Skeat and Blagden (1906). They named these groups of people as the “woolly-haired” Negrito, “wavy-haired” Sakai, or “straight-haired” Jakun. Nonetheless, disagreements remained, and new competing theories continued to be proposed. Some did not agree with the separation between Negrito and Sakai (Annandale and Robinson, 1902); and one distinguished them into: Semang, the Northern Sakai, the Central Sakai, the Bersisi, and the Jakun (Wilkinson, 1926). The tripartite classification of Negrito, Senoi, and Proto Malay, was only firmed in the 1930s. Subsequent sub-tribes of the Orang Asli populations were categorized based on their linguistic dialects.

The Negritos from the SEA was once argued to be the remnants of the wrecked slave ship from Africa (Crawfurd, 1820), yet the possibility that they had a much deeper link hinting at the earliest inhabitant in SEA was not refuted. The connections between these “eastern” Negrito (as labeled by Crawfurd) and Africa became a hot topic of argument. The alternative Pan-Negrito theory was proposed, which argued that all indigenous people of Malaya were Negrito origin and were linked to others with Negrito physical characteristics, but was rejected (Skeat and Blagden, 1906). In the late 1930s, the role of SEA Negritos in human migration history was heavily debated, and the idea pertaining the connections between the African and Asian “pygmies” were revived among the anthropologists (Endicott, 2016).

The population classification for the Bornean native populations has been far more complicated because most of these populations have indistinguishable physical appearance and economic activities; yet a rich variety of cultural heritage and linguistics. These populations were classified as the East Asian populations, as exemplified by several native populations in the Philippines and Taiwan (Williams, 1965). The classification of the Bornean populations is more complex hence inconclusive and still under debate to date.

The distinct phenotypic characteristics of these SEA inhabitants along with a handful of archaeological and linguistic evidence thence provided hints to the early “Two-Layer” migration hypothesis, which argued that SEA was occupied (the first layer) by the direct descendants of the early modern humans out-of-Africa, and subsequently admixed with the later immigrants (the second layer) from North and/or East Asia leading to the present day SEA human diversity (Bellwood et al., 2007; Matsumura et al., 2008).

### Blood Groups Markers

It was not until the 1950’s that a new form of anthropological measurement was introduced into the consideration of the “race” classification – the blood group (Table 2). The study for blood group anthropology was claimed to be the “first generation of human population genetics” (Endicott, 2016).

**TABLE 2:** The proportion of blood group and population classification based on Biochemical Racial Index.

Blood group	Population
Proportion higher of “A”	European
Proportion higher of “B”	Asio-African
Equal distribution of “A” and “B”	Intermediate type of population
Proportion higher of “O”	Island folk; isolated population

The first known blood group test on Orang Asli was carried out in the 117 Semai sub-tribe, which showed a higher frequency of the “O” blood group (Green, 1949). This report had led to a postulation that the Semai was an isolated population and somehow connected to other indigenous populations such as Tho and Muongs in mainland SEA and Tobias in Sumatera.

**FIGURE 2:**
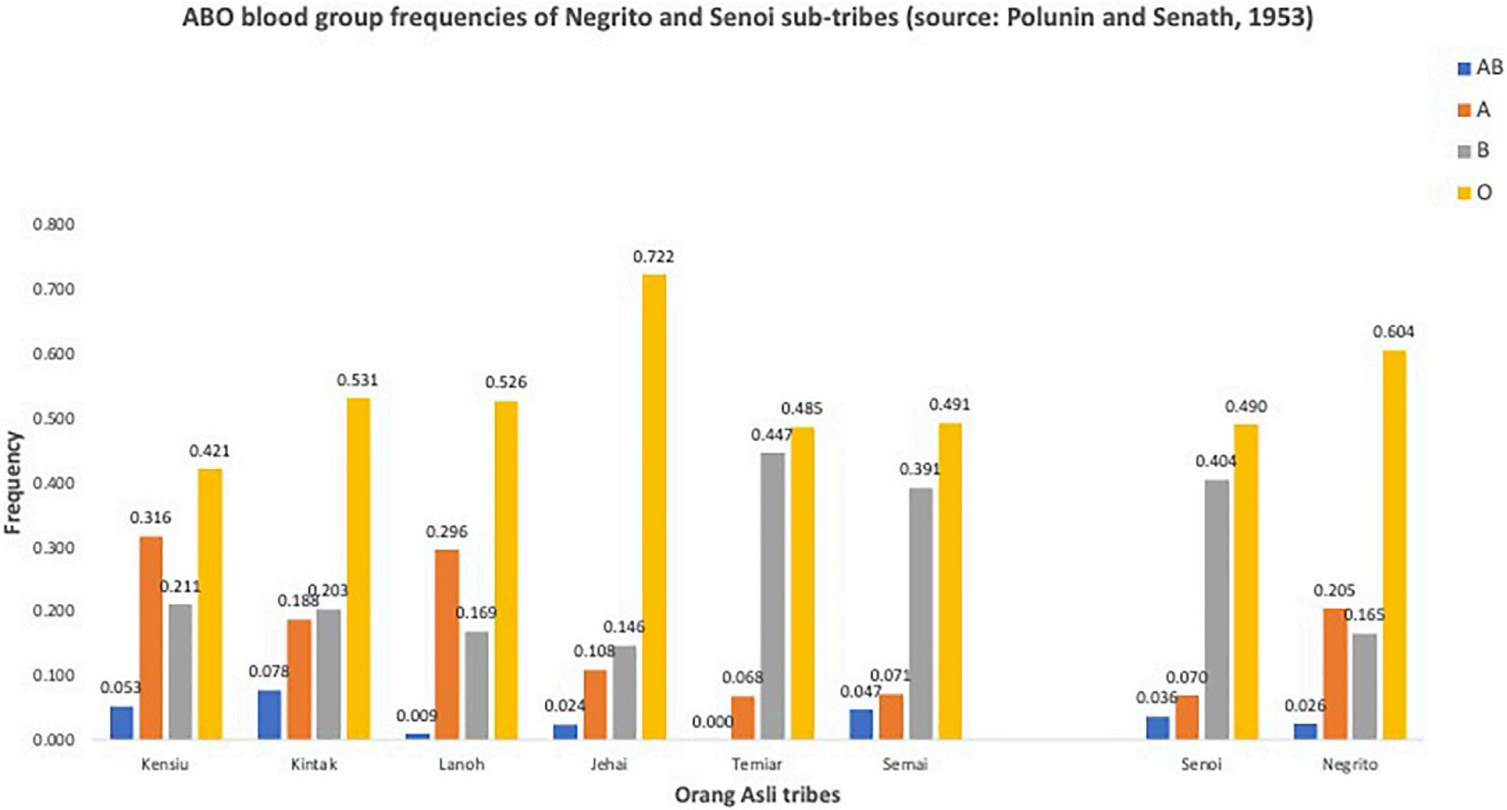
ABO blood group frequencies of Negrito and Senoi sub-tribes (adapted from: Polunin and Sneath, 1953).

Further investigations into blood groups were carried out, attempting to address some contentious questions like classifications of the Orang Asli; relationships between the Orang Asli Negrito with the Negritos from Africa, SEA, and Australia, and the links between the Orang Asli groups with the rest of the populations in the world. Many of these studies were carried out by Ivan Polunin (Polunin, 1953; Polunin and Sneath, 1953). The categorization of the populations was primarily based on the tripartite classification and was very much relied on the physical anthropological measurements. The study showed that the populations classified under Negrito had lower frequency for the B blood group compared to those classified as Senoi (Figure 2), and noted similarities between the Senoi ABO frequencies to those people from India and Burma. On the other hand, the complete absence of sickle cell trait in Negrito, and the frequency for B blood group in the native Iban in Sarawak that was found similar to the Negrito (Graydon et al., 1952; Tan, 1978), had posted doubts on their connection to the African ancestry.

### Protein Markers

The period between the 1960s–1980s marked the advancement of population genetics with the popularity of protein electrophoretic and isozyme variations from erythrocytes, serum, cerumen, human placenta, and saliva. The majority of these works were carried out by Lie-Injo Luan Eng and Tan Soon-Guan (Lie-Injo and Chin, 1964; Luan Eng, 1965; Lie-Injo, 1969; Steinberg and Eng, 1972; Lie-Injo et al., 1976; Tan, 1978; Tan, 1979; Tan and Teng, 1978; Tan et al., 1982) (Supplementary Table S1). Some findings worth acknowledging: 1) Gamma globulin variant (Gm) in Northern Orang Asli (presumably the Negrito) varied markedly from other Orang Asli groups; 2) Temiar and Semai had similar Gm variant frequency; 3) some malaria-related genes from the Negritos were different from the Southern Orang Asli (presumably the Proto Malays); 4) gene variant ADA-2 (Adenosine deaminase) and Pep B-6 (Peptidase B) in Semelai were similar to Temuan; 5) analysis based on five isozyme markers indicated that Sabahan native Kadazan was closer to their neighboring population as well as the Taiwanese and Philippine aborigines. In general, these biochemical markers were able to distinguish the East Asian from the non-East Asian groups of populations. Among the East Asian cluster, the Proto-Malay was found to cluster with the indigenous populations from Taiwan; whilst the natives from Sarawak formed another cluster (Tan, 2001).

Other intriguing observations included the frequencies Hb E, G6PD deficiency, and ovalocytosis among the native populations from Malaysia that were parallel with past distributions of malaria posted the theory that malaria is selective for these hematological traits (Luan Eng, 1965; Lie-Injo, 1969).

Although the questions pertaining to the history of peopling and migrations in Malaysia and SEA were not noticeably addressed in these early studies, the clarification of population relationships using these conventional technologies has essentially laid an important foundation for future population genetics, or subsequent studies in Malaysia could not have been made successful.

### Mitochondrial and Autosomal DNA Markers

The availability of molecular markers in particular maternal lineage mitochondrial DNA (mtDNA) markers, and subsequently molecular clocking approach have completely changed the way we appreciate the human migration and peopling history in Peninsular Malaysia. Several postulations on the migration history of SEA were initially proposed but posited considerable doubts. For instance, Ballinger et al. (1992) proposed a genetic continuity of Southern China migration of the Orang Asli, but the samples classification was questionable (Baer, 1999). In other studies, genetic trees produced by Cavalli-Sforza et al. (1994) and Saha et al. (1995) were inconsistent with the accepted historical events (Endicott, 2016), leading to an inconclusive genetic history of Orang Asli. Fix (1995) on the other hand, illustrated the introduction of an autosomal adaptive allele of SEA ovalocytosis (SAO) to the Orang Asli from the islands instead of mainland SEA, therefore postulated that the present Orang Asli is a product of local acculturation and differentiation.

Despites the said controversial, several hallmark studies had provided further insights into the prehistorical peopling in SEA. Based on mtDNA variation, Macaulay et al. (2005) suggested a seminal publication, that there was a single dispersal of the modern humans out-of-Africa ∼65 kya, migrated along the southern coastal route through present-day India towards SEA and Australasia. Hill et al. (2006) claimed that the Orang Asli Negrito carry deep ancestry haplogroups dating to the initial settlement out-of-Africa more than 50 kya; whereas half of the maternal lineages of Senoi traced back to the ancestors of the Negrito and the remaining to Indochina, supporting the postulation that they represent the descendants of early Austroasiatic speaking migrants. The Proto Malay were more diverse and had closer affinity to the populations from the islands of SEA. These studies found that the ancient haplogroup M21a and R21 was most frequent among the Negrito and Senoi; whilst N21 and N22 that are thought to be specific to SEA, largely occurred in the Proto Malays. The M21 haplogroup branches directly from the Eurasian founder haplotypes, hence implied that the Negrito is probably the most direct descendants of the original inhabitants of the Malay Peninsula. Their findings also suggested that there were at least four migration events that shaped the genetic make-up of the indigenous populations in Peninsular Malaysia. The fact that the Senoi carried the Indo-Chinese haplogroup F1a1a and N9a6a (date ∼5,500 years ago), suggested that they could have been descended from the admixture of local hunter-gatherers with the later agricultural invaders (Hill et al., 2006; Oppenheimer, 2011).

### Whole Genome Analysis

The completion of the human genome project has revolutionized and accelerated the progress of population genetic studies at a tremendous pace. Genome-wide genotyping array (SNP array) and next-generation sequencing technologies were subsequently made available for large genome mapping initiatives to be carried out, including Human Genome Diversity Project (Cavalli-sforza, 2005), the International HapMap Project (The International HapMap Project, 2003), and the more recent 1000 Genomes Project (The 1000 Genomes Project Consortium, 2010). Genotyping on the modern Malay populations were carried out (Teo et al., 2009), followed by large scale whole genome sequencing (Wong et al., 2013; Wu et al., 2019).

Surprisingly however, the indigenous populations from SEA were underrepresented in these large initiatives. Acknowledging this notion, the HUGO Pan-Asian SNP (PASNP) Consortium initiated the mapping of the Asian human genetic diversity with ∼50,000 genome-wide autosomal SNPs, and revealed that the ancestry of Asian populations harbors genetic contributions derived from five language groups, and that the migrations of SEA populations were more complex than the anticipated. Most notably, the study supports an initial single migration wave of the ancestors of East Asian (EA) populations via “Southern route” into SEA, followed by multiple subsequent migrations thence shaped the complex genetic diversity of SEA (HUGO Pan-Asian SNP Consortium et al., 2009). The groundbreaking findings have placed the Orang Asli as an important piece of puzzle in mapping modern human migration.

Subsequently a growing body of evidence thenceforth refined the peopling history in Malaysia. The SEA Austronesian populations generally carried ancestry components derived from four primary admixture events: 1) aboriginal Taiwan; 2) Austroasiatic; 3) Melanesian; and 4) Negrito (Lipson et al., 2014); whilst the western islands of SEA populations was found to carry the ancestral components originated from the present-day mainland SEA Austroasiatic populations (Hudjashov et al., 2017). This migration model is consistent with the alternative “Early Train” migration hypothesis proposed by Jinam et al. (2012), which argues that there was a migration originating from Indochina or South China ∼30–10 kya. Both hypotheses are supported at least in part, by several other lines of evidence: 1) close genetic affinity between the Sabahan natives and the Taiwanese aborigines and the Philippines aborigines but distantly related to the populations from mainland SEA (Yew et al., 2018a); 2) putative signals of positive selection driven by malaria infection found in the Sabahan natives occurred ∼5 kya, which coincides with the period during Austronesian expansion (Hoh et al., 2020); 3) inference divergence time between the Negrito and Senoi coincides with the proposed period of “Early Train” hypothesis, posing the plausibility of the swiddening Austroasiatic agriculturist migration to Peninsular Malaysia, which resulted in declined effective population size of Negrito (Yew et al., 2018b); 4) inference using both uniparental and autosomal markers suggested primarily common ancestry for Taiwan or islands of SEA populations established before the Neolithic period (Soares et al., 2016); 5) the native from Sarawak (the Iban) showed a closer genetic affinity to Indonesia than the mainland SEA (Simonson et al., 2011).

**FIGURE 3:**
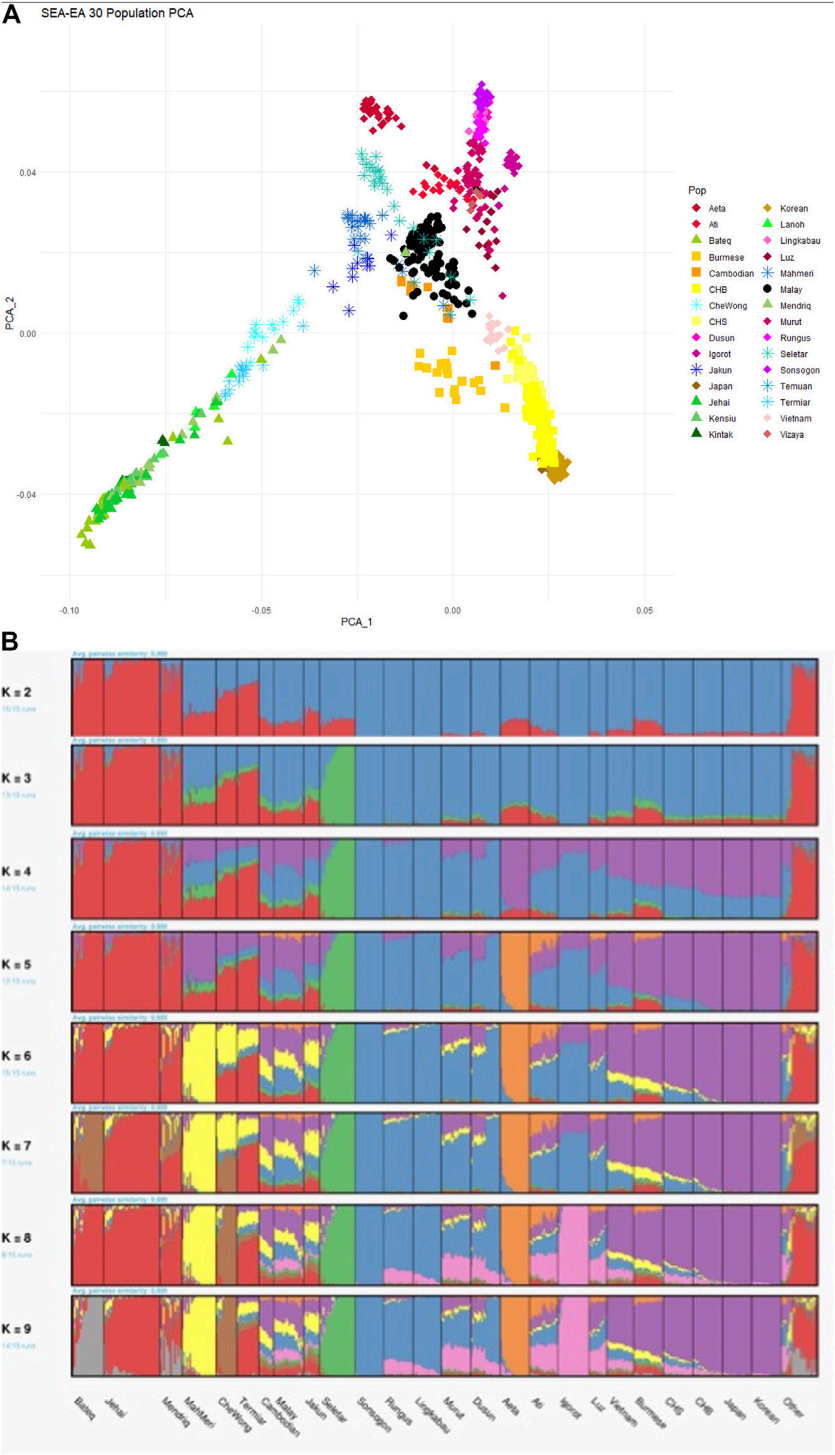
PCA and ADMIXTURE analysis between the Malay (MAS) and HGDP-Cambodian populations. **(A)** Both MAS and HGDP-Cambodian are clustered close to each other in PC1 and PC2. **(B)** In ADMIXTURE analysis, the ancestral components for the MAS and HGDP-Cambodian are indistinguishable.

When dissecting the genetic architecture of the modern Malays from Peninsular Malaysia, population substructure was observed, suggesting plausible different origins from neighboring regions of SEA (Hatin et al., 2011). Finer investigation suggested that the modern Malays substructure is clustered to Northern and Southern Peninsular, correlated with the geographical latitude of the respective sampling locations (Hoh et al., 2015). The ancestors of the modern Malays diverged from the East Asian ∼25 kya; but subsequently admixed with the other Austronesian populations ∼1,700 years ago (Wu et al., 2019). This finding is in part consistent with our earlier study, which identified four major admixed ancestral components in the Malay populations occurred ∼1,500–750 years ago: Austronesian, Proto Malay, East Asian, and South Asian (Deng et al., 2015). The slight violation of the admixture time between our study as opposed to Wu et al. (2019) is likely due to the different experimental platforms acquired. The collective findings suggest that geographical isolation and independent admixture have significantly shaped the genetic architectures and the diversity of the present days modern Malays. Intriguingly, the Malays revealed a close genetic affinity to the Cambodians (from the Human Genome Diversity Project dataset) (Deng et al., 2015; Liu et al., 2015) (Figure 3). The underlying reason to this link is uncertain, thus warrants further investigations.

The notion of multiple prehistorical migration waves to SEA was further supported by ancient genomes analyses (Lipson et al., 2018), revealing that the early farmers from Indochina (Vietnam) carried an admixed genetic component of East Asian (southern Chinese agriculturalist) and deeply diverged eastern Eurasian Austroasiatic-speaking hunter-gatherer ancestry, with similar ancestry south Indonesia, thus supporting an initial expansion of Austroasiatic languages.

Whilst the peopling history of Austronesian is seen clearer, the genetic affinity between the Orang Asli Negrito and ancestors of the Austroasiatic language-speaking populations remains controversial. Growing body of evidence confirm that the Orang Asli Negrito once shared common origins with the East Asian populations (Deng et al., 2014; Aghakhanian et al., 2015; Liu et al., 2015; Fu et al., 2018; Yew et al., 2018a). However, on a finer scale, they are distinct from the rest of the East Asian populations (Figure 3). The inference of divergence and admixture time using autosomal SNPs supports the argument that Negrito as the descendants of the earliest inhabitant of SEA (Deng et al., 2014; Aghakhanian et al., 2015; Yew et al., 2018a). Although multiple archaeological and mtDNA evidence suggested deep ancestry lineage 50–60 kya in the Orang Asli Negrito, their inferred time of divergence from Eurasian was younger than anticipated (∼31–50 kya) (Jinam et al., 2017; Yew et al., 2018a). However the findings were consistent with the divergent time for Andamanese Negrito (∼50 kya) (Mondal et al., 2016) and the northern Philippines Negrito (∼46 kya) (Larena et al., 2021b). Intriguingly, despites exhibiting similar phenotypic characteristics, the Negritos from Malaysia, the Philippines and the Andaman were found to be distantly related. On the other hand, an ancient link between the Negrito from Peninsular Malaysia and the Andamanese Negrito was observed (Aghakhanian et al., 2015), but the proportion was too weak to confirm it significance. Our recent investigation successfully revealed a specific basal Asia ancestry component exclusively shared by the Negrito populations from Asia, dated at least 50 kya (Deng et al., 2021). Collectively, it is conceivable to speculate that the ancestors of these Negrito populations could have split prior arriving to the Sundaland, subsequently entered the mainland- and islands- of SEA independently, eventually remained isolated and formed genetic architecture unique from each other.

Intriguingly, the Orang Asli Negrito (Jehai sub-tribe) showed a shared genetic drift with ancient genomes from Hoabinhian ancestry (McColl et al., 2018), suggesting that they are genetically closer to the ancestors of Hoabinhian hunter-gatherers who occupied northern parts of Peninsular Malaysia during the late Pleistocene (Bellwood, 2007). What puzzles us, however, is that the divergence between Eurasian and Australian aborigines (Malaspinas et al., 2016) predates the divergence between Eurasian and Orang Asli Negrito (Yew et al., 2018a), thus complicates the peopling history in Peninsular Malaysia.

Recent investigation postulates that pre-Neolithic ancestors of today’s Austroasiatic language-speaking populations were widespread in South Asia and SEA, and the populations from these regions were the result of multiple migrations of East Asian farmers during the Neolithic period (Tagore et al., 2021). The authors predicted that the present-day Austroasiatic populations from India and Malaysia shared a common ancestor until about 10.5 kya. Post-separation, they had a disparate genetic history. Around 7 kya, with the agriculture expansion, there was an ancestry shift in SEA. While the study has reiterated the impact of Austroasiatic populations in Peninsular Malaysia, how and when did the ancestors of Austroasiatic and Orang Asli Negrito introgressed was not addressed.

We acknowledge however, some of these findings should be interepreted with caution, because many of these studies were carried out with a rather small sample size, which may have introduced sampling biasness hence compromised conclusion.

### Archaic Genomes Analysis

Whilst some old questions are addressed, peopling history in SEA is complicated by the genomic introgression between modern human and archaic hominin. Denisovan genome introgression was found significantly higher in the Papuan and the Philippine Negrito (Mamanwa); but not in the native populations from Peninsular Malaysia Borneo, and the Andamanese Negrito (Reich et al., 2011; Jinam et al., 2017; Yew et al., 2018a). Therefore it was speculated that the introgression could have occurred in the common ancestors of Australian aborigines and Papuans and the Philippines Negrito, before the divergence of with these populations and the Orang Asli Negrito; and that the similar proportion of introgression between SEA and East Asian is likely the ancestral component (Yew et al., 2018a). The recent report revealing that the Philippine Ayta carry the known highest level of Denisovan ancestry in the world – 30–40% greater than that of Papuans – therefore supports the view on an independent admixture event into the Philippine Negritos from Denisovans. The Philippine region is thus likely inhabited by multiple archaic groups prior to the arrival of modern humans (Larena et al., 2021a), which again prolongs the debate on the links between the Orang Asli Negritos and the Negritos from the Philippines, Australia, and the Papuans. Considering that Denisovan introgression in the Philippine Negrito and Papuans could have occurred independent of the Orang Asli Negrito, it again supports the postulation that these phenotypically Negrito-like populations may have occupied the SEA landmass via different migration waves.

What is more intriguing, is that genome analysis of a human remained dated ∼7 kya surprisingly expressed a substantial East Asian ancestry component, with a mixture of Denisovan gene flow, indicating an East Asian ancestry present in Wallacea much earlier than Austronesian expansion. This discovery has overthrown our common understanding of SEA human peopling history, thence shows that the peopling of SEA was much more complex than has previously been appreciated (Carlhoff et al., 2021), and the natives from Peninsular Malaysia and Borneo may be an important key to this answer.

The cumulative evidence presented above thenceforth implies “multi-layer” migration to SEA, and Peninsular Malaysia and Borneo to be more specific (Mörseburg et al., 2016), instead of the simplified “two-layer” migration model.

For the ease of reference, a summary of the published genotyping and sequencing data for the natives of Peninsular Malaysia and Borneo is tabulated in Supplementary Table S2.

### What Is Known, and What Is Unknown?

With the advent of genomic technology, many long-standing questions were addressed with renewed vigor, some possibly with refined insights. Based on the collective findings to date, the following section summarizes, what we know about the peopling and migration history of the native populations from Malaysia:

(i) The ancestors of SEA populations migrated out of Africa via “Southern Route” along the coastal line to East Asia (Hill et al., 2006; HUGO Pan-Asian SNP Consortium et al., 2009).
(ii) The Negrito from Peninsular Malaysia once shared a common ancestry with East Asian populations (HUGO Pan-Asian SNP Consortium et al., 2009; Aghakhanian et al., 2015; Liu et al., 2015; Jinam et al., 2017; Lipson et al., 2018).
(iii) Both mtDNA molecular clocking and inference of divergence time using autosomal DNA support the notion that the ancestors of Peninsular Malaysia Negrito may be the earliest inhabitant of SEA at least 50 kya (Hill et al., 2006; Yew et al., 2018a; Deng et al., 2021).
(iv) The native populations from Peninsular Malaysia and Borneo are genetically distinct, each with a unique population history (Yew et al., 2018b).
(v) Austronesian expansion occurred at least in the SEA region, southwards to the Philippines, towards the Northern Borneo (Hill et al., 2007; Lipson et al., 2014; Yew et al., 2018b).
(vi) There are at least four prehistorical gene flows events that occurred in Peninsular Malaysia and Borneo thus concurs the “multi-layer” migration model. The first migration was likely to had derived from the earliest ancestors of the Negrito-like populations; subsequently the Austroasiatic migration southwards replacing the descendants of the early inhabitants. The third migration being the Austronesian expansion, and the last likely being the East Asian or islands of SEA (Hill et al., 2006; Jinam et al., 2012; Lipson et al., 2014; Deng et al., 2015; Soares et al., 2016; Mccoll et al., 2018; Lipson et al., 2018).
(vii) The native populations from both Peninsular Malaysia and Borneo received minimal gene flow from the archaic Denisovan hominin (Reich et al., 2011; Yew et al., 2018a).

**FIGURE 4:**
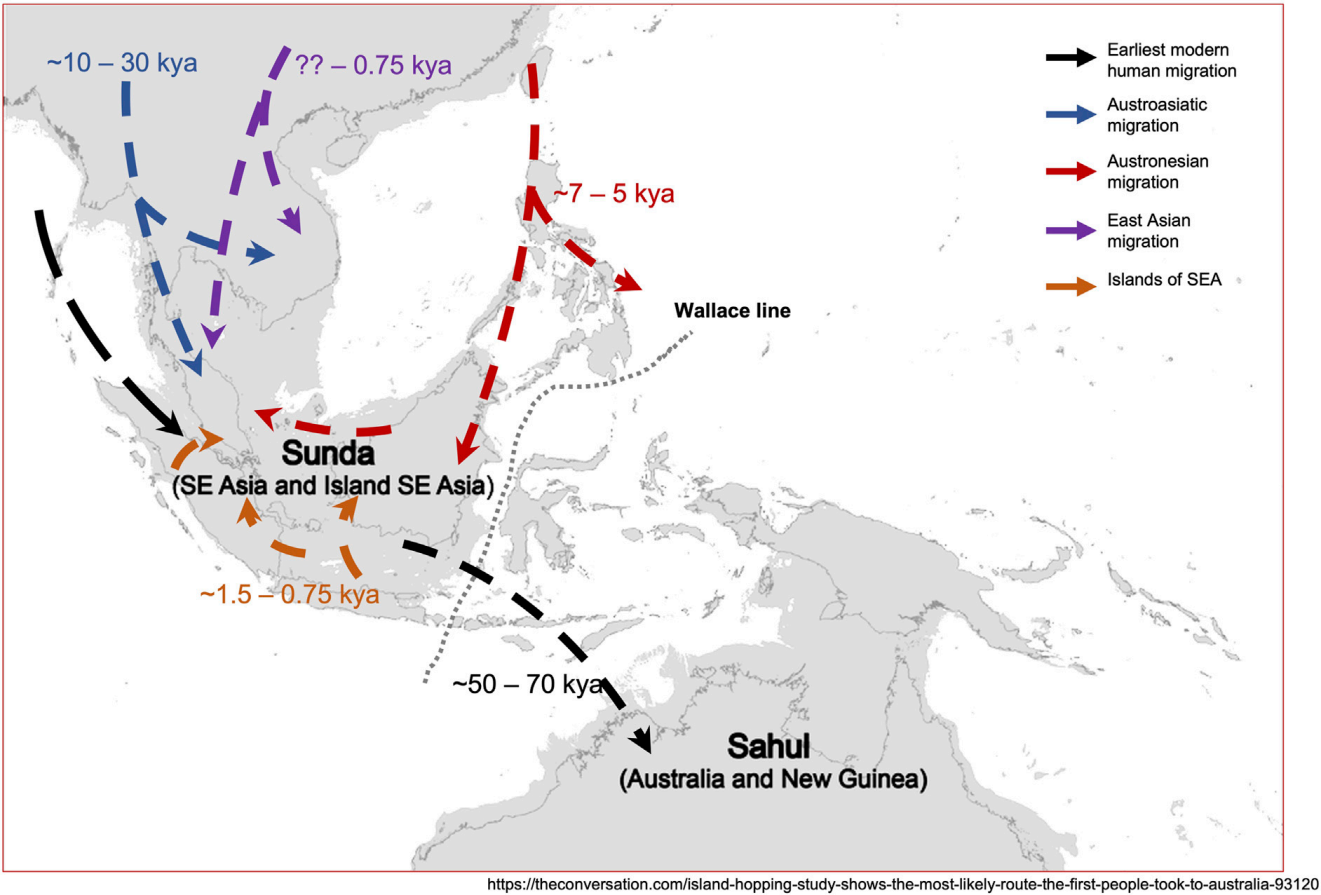
A hypothetical model of migration history of the native populations from Malaysia. The map was adapted and modified from https://theconversation.com/island-hopping-study-shows-the-most-likely-route-the-first-people-took-to-australia-93120, with permission.

Based on our current understandings along with earlier proposed models (Lipson et al., 2014; Norhalifah et al., 2016), we redraw a hypothetical modern human migration routes into Peninsular Malaysia (Figure 4).

What remained to be answered, we think, are:

(i) The genetic link between the Negrito-like populations from SEA, including Negrito from Peninsular Malaysia, the Philippines, the Andaman, Papuan and Australian aborigines, and possibly the African pygmies, remain inconclusive. Whilst it has been shown that these populations shared an ancient basal Asian ancestry component (Deng et al., 2021), they have undergone a long period of isolation from each other and could have experienced extensive local adaptation and admixture from respective neighboring populations therefore posting a challenge to uncover the precise date of migration of these populations. Analysis of natural selection may be able to at least in part address this question (Liu et al., 2015; Zhang et al., 2021). Several studies have been carried out in the SEA populations including the natives from Peninsular Malaysia and Borneo (Liu et al., 2015; Liu et al., 2017; Hoh et al., 2020). However finer genotyping, phenotypic and environmental characteristics, along with a sample size on the indigenous populations are required to warrant a convincing conclusion.
(ii) The pre-historical migrations of the Negrito into this landmass remain fragmentary. If the Orang Asli Negrito were postulated to be the direct descendants of the modern human out of Africa and that the divergence time between Australia aborigines and Eurasia occurred 65 kya (Malaspinas et al., 2016; Clarkson et al., 2017), we would then anticipate the divergence time between Orang Asli Negrito and Eurasia to have overlapped with the Australian aborigines if not earlier. However, our inferred time of divergence was younger than the Australia aborigines-Eurasia divergence time. Comprehensive investigations on a high coverage of SEA Negrito populations along with their neighboring populations are required before a conclusion can be made.
(iii) What are the relationships between the Orang Asli Negrito and Senoi populations? Different postulations have been made on the origins of Senoi. Some related them to the Veddoids; others argued that they were the descendants of admixture between Negrito and Austronesian (Tan, 2001; Oppenheimer, 2011). However, our preliminary PCA shows that the Senoi subtribes are much more heterogenous than the Negrito (Figure 2), suggesting that there is no simple package of elements to this conclusion. The inference of divergence time earlier proposed that they may be the descendants of the swiddening Austroasiatic agriculturists who later occupied Peninsular Malaysia (Yew et al., 2018a). Dissecting the genetic structure of the Senoi, and subsequently the connections between the Negrito and Senoi would be challenging owing to their close genetic affinity.
(iv) Reason(s) of the absence of Negrito-like populations in Borneo. Archaeological evidence suggests that Borneo was first inhabited by the ancestor of SEA modern human who were believed to be the direct descendants from the out of Africa exodus (Reyes-Centeno et al., 2015; Curnoe et al., 2016). It was postulated that these populations were then replaced by the Austronesians (Bellwood, 2007). If it was true, then how and when did it happen?
(v) It is intriguing to observe a close genetic affinity between the modern Malays and Cambodian population (but on the Proto-Malay Jakun). Further investigation should be carried out to explain the links between the modern Malay, Cambodian and Yunnan Migration Hypothesis; or to dispute this hypothesis.
(vi) Soares et al. (2016) argued that gene flow could have happened from the islands of SEA to Taiwan and subsequently back-migration again occurred. This argument does not agree with the common understanding of “Out-of-Taiwan” expansion as supported by linguistic and archaeological evidence. They subsequently claimed that there were two minor gene flows from Taiwan rather than a massive migration waves. In addition, recent study claimed no strong support for a predominant out-of-Taiwan dispersal of rice (Larena et al., 2021b), suggesting the “Out-of-Taiwan” model may be more complex than expected. More thorough investigations using both uniparental and high-density autosomal markers involving comprehensive native populations from SEA and Polynesian are required to conclude the Austronesian migration model.

### Challenges of Studying the Population Genetics of Natives in Malaysia

The population genetics study in Malaysia is not without challenge. Owing to their long period of isolation and high inbreeding rate, population genetic studies of the native populations – specifically referring to the Orang Asli and the Bornean natives – are often restricted to limited number of unrelated samples. This hinders the statistical power thence affects the strength of the evidence to support or dispute a hypothesis, resulting in a rather limited impact publication. In addition, although the cost for genome-wide analysis has reduced dramatically, it is still unaffordable for many low- and middle-income countries. Limited research funding available for such less prioritized science has always been a challenge to carry out high-throughput genome sequencing initiatives.

Nonetheless, the more pressing challenge is the ethical and integrity concerns that have been raised over the years, particularly issues on accessing the samples and data. A thorough process of ethics approval has been established especially for the genetic study of native populations in SEA countries, such as Malaysia, the Philippines and Indonesia. Efforts are spent in engaging and building trusts with the native populations, and collecting biological materials and data. Often such a process takes years before seeing success. Therefore, the contribution from the local scientists merits scientific acknowledgments. Unfortunately, we see occasionally, scientific publications or reports on the marginalized populations from SEA without appropriately acknowledging the local scientists. Such practice has raised some arguments over the years. We, therefore, call for close collaborations between investigators locally and abroad to warrant the advancement of science in mutual respect and ethical manner.

## The Implications of Migration and Peopling History of SEA Populations on Human Health and Diseases

The complex demographic history of modern humans out-of-Africa has created different genetic architectures across populations from different continents, hence disease susceptibility. With genomic data from diverse populations and ancestries, we can compare these information over time and geography to better understand the origins and evolution of both individual genetic variants and human populations.

From global perspective, studying the genomics of the SEA marginalised populations, in this particular case, the natives from Peninsular Malaysia and Borneo, complements the catalogue of genetic variations that is known to be strikingly bias to European populations. Particularly, it allows characterizations of rare, and population (or ancestry) specific genetic variations (Bustamante et al., 2011; Hindorff et al., 2018). A classic success story of studying the genomic of natives is the Greenlandic Inuit populations, which found strong signals of adaptation to diet rich in protein and fatty acids (Fumagalli et al., 2015; Zhou et al., 2019). Another example is the Madagascar population (Pierron et al., 2018). Analysing the admixture and local ancestry of this population revealed a strong selection signal underlying Duffy blood group gene (*FY*) against *Plasmodium vivax* infection, and the selection signal was derived from the Asian ancestry.

From regional perspective, many tropical diseases that are uncommon in many developed countries, for instance Dengue and nasopharyngeal carcinoma. In addition, owing to different demographic history, climate, diet and lifestyles, pathophysiology for many complex diseases, for instance, cardiovascular diseases and metabolic syndromes, in the SEA populations may vary from the developed countries, and manifest in different intermediate phenotypes. Therefore, population genomic studies of the native will be utmost pertinent to the disease mapping efforts in SEA.

## Conclusion

The population genetic study of the native populations in Malaysia has begun since the 19th century with the intention of racial classification. Postulations and arguments on human migration and peopling history especially to the SEA region were merely based on anthropology, archaeological, and linguistics evidence. With the advancement of genomics and life science technology, the picture of the peopling and migration history of the natives from Malaysia, and their attributions with other neighboring populations has become clearer. The natives from Malaysia were originated from multiple waves of migration events. It began with the direct descendants of the exodus of out-of-Africa, followed by the early Austroasiatic primitive farmers from Indochina that occupied the Peninsular Malaysia region and interacted with the existing inhabitants; subsequently with multiple waves of migrations of Austronesian populations from Southern China (and Taiwan) and other islands of SEA. However, we acknowledge that the migration model is far more complex than anticipated, and there are doubts yet to be addressed. We reiterate here, the importance of unveiling migration history and genetic diversity of the indigenous populations, not only to address the fundamental question of the origin of modern humans, but to complement the catalog for human genome variation, and to provide a stepping stone towards comprehending disease evolution and physiology (Fan et al., 2016; Hindorff et al., 2018; Wu et al., 2019). Increased attention to diversity will eventually increase the accuracy, utility and acceptability of using genomic information for clinical care (Benton et al., 2021).
