# Supplementary material for: The Peopling and Migration History of the Natives in Peninsular Malaysia and Borneo: A Glimpse on the Studies Over the Past 100 years
Source: Front Genet. 2022 Jan 27;13:767018. doi: 10.3389/fgene.2022.767018 (PMC8829068; doi:10.3389/fgene.2022.767018)
Supplement: Supplementary file 1 [file DataSheet1.docx]

**SUPPLEMENTARY TABLES:**

**Table S1.** Summary of the major findings based on the protein markers.

| **Reference** | **Major findings** |
| --- | --- |
| (Lie‐Injo and Chin, 1964) | This study found unusually high HbE frequency among the Temiar (Senoi subtribe) (~47%); whilst ~23% of the Semai and Jahut (both Senoi subtribes) were G6PD deficient, posting the possibility of correlation with high malaria prevalence among the indigenous populations. |
| (Lie-Injo Luan Eng, 1965) | High prevalence of ovalocytosis was found among the Senoi (12.3% as opposed to previously estimated 0.04% in general population) |
| (Lie‐Injo et al., 1967) | This study found unusually high frequency of ahaptoglobinaemia among the Orang Asli, however there was no supporting evidence to the attribution with malaria, HbE and G6PD deficiency.  The serum gamma globulin gene variation pattern was different from other populations including Caucasian, East Asian, other Negrito populations and Australian aborigines. |
| (Eng, 1969) | A review on the distributions of the red cell related traits in the Southeast Asia populations, including HbE, Thalassaemia, and G6PD deficiency. Cumulative evidence supported the attribution of malaria to the distributions of these red cell related defects. However this review did not refute the human migration history as a plausible factor that influenced the frequencies of these defects. |
| (Steinberg and Eng, 1972) | This study investigated the genetic variants of immunoglobulin G (Gm) frequencies among the Semai (Senoi subtribe) and Negrito, and found that the Negrito had higher frequency of Gm^1,21^ phenogroup compared to other indigenous populations and the African pygmies. This study claimed that the Melanesian and Micronesian populations shared similar Gm phenogroup frequencies with the indigenous populations in Peninsular Malaysia, thus speculated that these populations once shared a recent common ancestry. |
| (Fix and Lie‐Injo, 1975) | This study investigated three genetic loci namely ABO blood group, hereditary ovalocytosis and haemoglobin among 546 Semai (Senoi subtribe) individuals sampled from seven locations, and found some degree of microdifferentiation among this population. The authors postulated reasons attributed to their respective migration and settlement histories, and possibly the recurrent founder effect. |
| (Teng et al., 1978) | The mitochondrial glutamic oxaloacetic transaminase (GOT) variation in Malay, Chinese and Indian showed a closer pattern to the Europeans with several 2-1 phenotypes. The study also found that the placenta phosphoglucomutase of the three populations studied (notably the Malay) showed similar PGM_3_ allele to that of the European populations. |
| (Tan et al., 1979) | This study found unusually high frequency of peptidase B (PEP B^6^) among the Sabahan Kadazan population as compared to Orang Asli, Australian aborigines, and the native from West Irian. |
| (Noraini et al., 1980) | This study investigated the salivary peroxidase (SAPX), Pm and Ph polymorphisms, but found no evidence of differences between the frequencies of these allele among Malays compared to other East Asian populations. |
| (Tan et al., 1981) | Group specific component (Gc) typing revealed that Gc^IF^ allele frequency was greater than the Gc^IS^ frequency for all Austronesian populations examined as opposed to the Indian population, which showed greater GC^IS^ frequency than the Gc^IF^ frequency. |
| (Tan et al., 1982) | This study investigated several major native populations from Borneo and Indonesia. The isozyme study on the Tranferrin C subtyping revealed rare frequency of Tf^C3^ among Bidayuh, Iban and Indonesian Minangkabau populations (<1%). No conclusion was made. |
| (Zarinah et al., 1984) | This study found that the ~25% of the Malays carried the uridine monophosphate kinase UMPK 2-1 allele, higher than the Chinese and Indians, as well as the North Borneo Kadazan (with the gene frequency 0.041), but lower than the Philippine Aetas (0.345). |

**Table S2.** Summary of the published genotyping and sequencing datasets for the natives from Peninsular Malaysia and Borneo.

| **Population** | **Platform** | **Accession no.** | **Reference** |
| --- | --- | --- | --- |
| Sarawak Iban | Affymetrix NspI (~250k autosomal SNP)  mtDNA  Y-chromosome | NA | (Simonson et al., 2011) |
| Modern Malay  Southern Chinese  Southern India | Illumina 1M (~1 million SNPs) | NA | (Teo et al., 2009) * |
| Modern Malay | Illumina whole genome sequencing | NA | (Wong et al., 2013)** |
| Southern India | Illumina whole genome sequencing | NA | (Wong et al., 2014)*** |
| Negrito Jehai  Proto Malay Seletar  Proto Malay Temuan  Sarawak Bidayuh | mtDNA sequencing | ﻿DDBJ/EMBL/Genbank (accession nos. AP012346–AP012431) | (Jinam et al., 2012) |
| Modern Malay | Illumina whole genome sequencing | Sequence Read Archive (SRA): PRJEB4210 | (Salleh et al., 2013) |
| Modern Malay (Kelantan)  Proto Malay Temuan  Senoi Temiar  Negrito Lanoh  Negrito Kintak  Negrito Jehai  Negrito Kensiu  Negrito Mendriq  Negrito Bateq | Affymetrix SNP6.0 (~900k autosomal SNPs) | NA | (Deng et al., 2014) |
| Negrito Bateq  Negrito Mendriq  Senoi Che Wong  Proto Malay Jakun | Illumina Omni2.5 (~2.5million SNPs) | National Omics Data Encyclopedia (NODE) (<http://www.biosino.org>): OEP000154 | (Liu et al., 2015) |
| Negrito Jehai  Senoi Mah Meri  Proto Malay Temuan  Proto Malay Seletar | Illumina Omni2.5 (~2.5 million SNPs) | NA | (Aghakhanian et al., 2015) |
| Modern Malays | Illumina 660W (~660k SNPs)  Illumina OmniExpress (~700k SNPs)  Affymetrix SNP6.0 (~900k autosomal SNPs) | NA | (Deng et al., 2015)  (Hoh et al., 2015) |
| Negrito Bateq  Negrito Jehai  Negrito Kintak  Proto Malay Temuan  Sarawak Bidayuh | Affymetrix SNP6.0 (~900k autosomal SNP) | NA | (Jinam et al., 2017) |
| Sabah Dusun  Sabah Murut  Sabah Lingkabau  Sabah Rungus  Sabah Sonsogon | Illumina Omni2.5 (~2.5 million SNPs) | ﻿National Omics Data Encyclopedia (NODE) (<http://www.biosino.org>): OEP000154 | (Yew et al., 2018b)  (Hoh et al., 2020) |
| Negrito Bateq  Negrito Mendriq  Senoi Semai  Sabah Murut  Sabah Dusun  Sabah Rungus  Sabah Sonsogon | Illumina whole genome sequencing | ﻿National Omics Data Encyclopedia (NODE) (<http://www.biosino.org>): NODEP00371760 | (Yew et al., 2018a)  (Deng et al., 2019) |

NA, not available

* ﻿<http://www.nus-cme.org.sg/SGVP/>

** <https://blog.nus.edu.sg/sshsphphg/singapore-sequencing-malay/>

*** <https://blog.nus.edu.sg/sshsphphg/singapore-sequencing-indian/>

**References:**

Aghakhanian, F., Yunus, Y., Naidu, R., Jinam, T., Manica, A., Hoh, B. P., et al. (2015). Unravelling the genetic history of Negritos and Indigenous populations of Southeast Asia. *Genome Biol. Evol.* 7, 1206–1215. doi:10.1093/gbe/evv065.

Deng, L., Hoh, B.-P., Lu, D., Saw, W.-Y., Twee-Hee Ong, R., Kasturiratne, A., et al. (2015). Dissecting the genetic structure and admixture of four geographical Malay populations. *Sci. Rep.* 5, 14375. doi:10.1038/srep14375.

Deng, L., Hoh, B. P., Lu, D., Fu, R., Phipps, M. E., Li, S., et al. (2014). The population genomic landscape of human genetic structure, admixture history and local adaptation in Peninsular Malaysia. *Hum. Genet.* 133, 1169–1185. doi:10.1007/s00439-014-1459-8.

Deng, L., Lou, H., Zhang, X., Thiruvahindrapuram, B., Lu, D., Marshall, C. R., et al. (2019). Analysis of five deep-sequenced trio-genomes of the Peninsular Malaysia Orang Asli and North Borneo populations. *BMC Genomics* 20, 842. doi:10.1186/s12864-019-6226-8.

Eng, L. L. (1969). Distribution of genetic red cell defects in South-east Asia. *Trans. R. Soc. Trop. Med. Hyg.* 63, 664–674. doi:10.1093/nq/s7-v.116.219c.

Fix, A. G., and Lie‐Injo, L. E. (1975). Genetic microdifferentiation in the Semai Senoi of Malaysia. *Am. J. Phys. Anthropol.* 43, 47–55. doi:10.1002/ajpa.1330430108.

Hoh, B.-P., Zhang, X., Deng, L., Yuan, K., Yew, C.-W., Saw, W.-Y., et al. (2020). Shared Signature of Recent Positive Selection on the TSBP1 – BTNL2 – HLA-DRA Genes in Five Native Populations from North Borneo. *Genome Biol. Evol.* 12, 2245–2257. doi:10.1093/gbe/evaa207.

Hoh, B. P., Deng, L., Julia-Ashazila, M. J., Zuraihan, Z., Nur-Hasnah, M., Nur-Shafawati, A. R., et al. (2015). Fine-scale population structure of Malays in Peninsular Malaysia and Singapore and implications for association studies. *Hum Genomics* 9, 16. doi:10.1186/s40246-015-0039-x.

Jinam, T. A., Hong, L., Phipps, M. E., Stoneking, M., Ameen, M., Edo, J., et al. (2012). Evolutionary History of Continental Southeast Asians : “Early Train” Hypothesis Based on Genetic Analysis of Mitochondrial and Autosomal DNA Data. *Mol. Biol. Evol.* 29, 3513–3527. doi:10.1093/molbev/mss169.

Jinam, T. A., Phipps, M. E., Aghakhanian, F., Majumder, P. P., Datar, F., Stoneking, M., et al. (2017). Discerning the origins of the negritos, first sundal and people: Deep divergence and archaic admixture. *Genome Biol. Evol.* 9, 2013–2022. doi:10.1093/gbe/evx118.

Lie-Injo Luan Eng (1965). Hereditary ovalocytosis and haemoglobin E-ovalocytosis in Malayan aborigines [25]. *Nature* 208, 1329. doi:10.1038/2081329a0.

Lie‐Injo, L. E., Bolton, J. M., and Fudenberg, H. H. (1967). Haptoglobins, Transferrins and Serum Gamma-globulins Types in Malayan Aborigines. *Nature* 215, 777.

Lie‐Injo, L. E., and Chin, J. (1964). Abnormal Haemoglobin and Glucose-6-phosphate Dehydrogenase Deficiency in Malayan Aborigines. *Nature* 204, 291–292.

Liu, X., Yunus, Y., Lu, D., Aghakhanian, F., Saw, W. Y., Deng, L., et al. (2015). Differential positive selection of malaria resistance genes in three indigenous populations of Peninsular Malaysia. *Hum. Genet.* 134, 375–392. doi:10.1007/s00439-014-1525-2.

Noraini, I., Tan, S. G., Gan, Y. Y., and Teng, Y. S. (1980). Salivary peroxidase, Pm, and Ph protein polymorphisms in Malaysians. *Hum. Genet.* 56, 205–207. doi:10.1007/BF00295695.

Salleh, M. Z., Teh, L. K., Lee, L. S., Ismet, R. I., Patowary, A., Joshi, K., et al. (2013). Systematic Pharmacogenomics Analysis of a Malay Whole Genome: Proof of Concept for Personalized Medicine. *PLoS One* 8. doi:10.1371/journal.pone.0071554.

Simonson, T. S., Xing, J., Barrett, R., Jerah, E., Loa, P., Zhang, Y., et al. (2011). Ancestry of the iban is predominantly Southeast Asian: Genetic evidence from autosomal, Mitochondrial, and Y Chromosomes. *PLoS One* 6, e16338. doi:10.1371/journal.pone.0016338.

Steinberg, A. G., and Eng, L. I. (1972). Immunoglobulin G allotypes in Malayan aborigines. *Hum. Hered.* 22, 254–258. doi:10.1159/000152495.

Tan, S. G., Gan, Y. Y., and Asuan, K. (1982). Transferrin C subtyping in Malaysians and in Indonesians from North Sumatra. *Hum. Genet.* 60, 369–370. doi:10.1007/BF00569221.

Tan, S. G., Teng, Y. S., Ganesan, J., Lau, K. Y., and Lie-Injo, L. E. (1979). Biochemical genetic markers in the Kadazans of Sabah, Malaysia. *Hum. Genet.* 49, 349–353. doi:10.1007/BF00569355.

Tan, S., Gan, Y., Asuan, K., and Abdullah, F. (1981). Gc Subtyping in Malaysians and in Indonesians from North Sumatra. *Hum. Genet.* 59, 75–76.

Teng, Y. S., Tan, S. G., Lopez, C. G., Ng, T., and Lie-Injo, L. E. (1978). Genetic markers in Malaysians: Variants of soluble and mitochondrial glutamic oxaloacetic transaminase and salivary and pancreatic amylase, phosphoglucomutase III and saliva esterase polymorphisms. *Hum. Genet.* 41, 347–354. doi:10.1007/BF00284769.

Teo, Y. Y., Sim, X., Ong, R. T. H., Tan, A. K. S., Chen, J., Tantoso, E., et al. (2009). Singapore Genome Variation Project: A haplotype map of three Southeast Asian populations. *Genome Res.* 19, 2154–2162. doi:10.1101/gr.095000.109.

Wong, L.-P., Ong, R. T.-H., Poh, W.-T., Liu, X., Chen, P., Li, R., et al. (2013). Deep whole-genome sequencing of 100 southeast Asian Malays. *Am. J. Hum. Genet.* 92, 52–66. doi:10.1016/j.ajhg.2012.12.005.

Wong, L. P., Lai, J. K. H., Saw, W. Y., Ong, R. T. H., Cheng, A. Y., Pillai, N. E., et al. (2014). Insights into the Genetic Structure and Diversity of 38 South Asian Indians from Deep Whole-Genome Sequencing. *PLoS Genet.* 10, e1004377. doi:10.1371/journal.pgen.1004377.

Yew, C., Lu, D., Wong, L., Twee-Hee Ong, R., Lu, Y., Wang, X., et al. (2018a). Genomic structure of the native inhabitants of Peninsular Malaysia and North Borneo suggests complex human population history in Southeast Asia. *Hum. Genet.* 137, 161–173. doi:10.1007/s00439-018-1869-0.

Yew, C. W., Minsong, A., Tiek, S., Lau, Y., Pugh-kitingan, J., Ransangan, J., et al. (2018b). Genetic relatedness of indigenous ethnic groups in northern Borneo to neighboring populations from Southeast Asia , as inferred from genome-wide SNP data. *Ann. Hum. Genet.*, doi.org/10.1111/ahg.12246. doi:10.1111/ahg.12246.

Zarinah, K. H., Abdullah, F., and Tan, S. G. (1984). Genetic markers in a malaysian population: Variants of uridine monophosphate kinase (UMPK), phosphoglycolate phosphatase (PGP) and pancreatic amylase (AMY2). *Ann. Hum. Biol.* 11, 533–536. doi:10.1080/03014468400007441.
